# A Manual of Human Physiology

**Published:** 1895-03

**Authors:** 


*A Manual of Human Physiology*. By JOSEPH H. RAYMOND, A.M., M.D. Pp. 382. Philadelphia: W. B. SAUNDERS. 1894.

This book contains a concise and clear epitome of the main facts of human physiology. It is written in a somewhat "popular" style, and gives the impression of the lecture-room rather than the laboratory.

The illustrations are numerous, and many of them are good, but their source is not always acknowledged as it should be: for instance, some of Maclise's plates, Horsley's and Schäfer's diagrams of cerebral convolutions, and some of Quain's excellent drawings are introduced with no mention of their authorship. We notice that the term "organic" is used to denote anything possessing "organs," and "proximate principles" as synonymous with "physiological ingredients." This nomenclature is puzzling, and liable to lead to confusion. Some of the figures given as to the percentage of water in urine, gastric juice, milk, &c., are inaccurate; but the mistakes are not numerous, and a great deal of care has been evidently taken in compiling the book and making it instructive. There are, however, so many excellent physiology books and primers now published in England, that we do not anticipate a large sale of the work in this country.

